# Selected Medicines That Can Cause Cardiac Arrest with Asystole

**DOI:** 10.3390/cimb47050299

**Published:** 2025-04-24

**Authors:** Kamila Czarnecka, Mateusz Jędrzejec, Aleksandra Kukiełczyńska, Jacek Owczarek, Łukasz Olejnik, Paweł Szymański

**Affiliations:** 1 Department of Pharmaceutical Chemistry, Drug Analyses and Radiopharmacy, Faculty of Pharmacy, Medical University of Lodz, Muszynskiego 1, 90-151 Lodz, Poland; mateusz.jedrzejec@umed.lodz.pl (M.J.); l.olejnik07@gmail.com (Ł.O.); 2 Department of Radiobiology and Radiation Protection, Military Institute of Hygiene and Epidemiology, Kozielska 4, 01-163 Warsaw, Poland; 3 Department of Pharmacology and Toxicology, Military Institute of Hygiene and Epidemiology, Kozielska 4, 01-163 Warsaw, Poland; aleksandra.kukielczynska@wihe.pl; 4 Department of Hospital Pharmacy, Faculty of Pharmacy, Medical University of Lodz, Muszynskiego 1, 90-151 Lodz, Poland; jacek.owczarek@umed.lodz.pl

**Keywords:** asystole, medicines, cardiac arrest, medication and asystole

## Abstract

One of the most serious consequences of cardiac arrest is asystole. It can occur in patients suffering from cardio-vascular diseases or during surgery following the use of certain drugs. The aim of this study was to identify the relationship between such use and the occurrence of cardiac arrest or asystole based on a review of literature identified in Science Direct, Web of Science and PubMed. Our findings confirm that a relationship exists between the use of certain drugs and the occurrence of asystole. Most drugs which induce asystole are used in cardiovascular disease, particularly beta-blockers, calcium L-channel blockers and potassium channel blockers. Medicine which can lead to asystole are drugs used, among others, for sedation during surgeries and intended for anesthesia; however, the relationship with asystole is not as clear as for the cardio-vascular drugs. Most patients who experience asystole during surgery after administration of the same drugs had other very serious health problems. Our findings are intended to support medical professionals in anticipating the possibility of asystole after drug administration.

## 1. Introduction

Asystole is a well-known type of cardiac arrest, where the heart loses excitability and thus stops pumping blood, characterized by cessation of electrical and mechanical heart activity. Asystole is often caused by drug resistant epilepsy [1], infarct, loss of a large amount of blood, vagal overactivity, cardiac arrhythmias such as ventricular tachycardia (VT) or ventricular fibrillation (VF), a defective pacemaker [2] and certain drugs such as beta-blockers and street drugs such as marijuana; it can also be caused by other chemical compounds that induce vagal and parasympathetic neuronal activity and by untreated bradycardia [3,4].

This type of cardiac arrest and cardiopulmonary resuscitation can cause pneumothorax, air embolism, spleen, stomach or colon rupture, hemothorax, liver laceration, fractured ribs, impaired cortical function and neurological complications. Asystole can also be manifested by seizure or syncope, and dizziness, which can appear six to eight seconds after the last heartbeat [5,6].

Recent decades have seen an increase in asystole and pulseless electrical activity (PEA) case numbers. They represent the most common causes of death within 24 h from cardiac arrest, with a greater risk of PEA and asystole being associated with male sex and a longer duration of cardiopulmonary resuscitation (CPR). A study conducted between 2000 and 2005 found that about 10.6% of patients in Edmonton, Canada survived one year after asystole. Importantly, patients with a shorter duration of CPR were more likely to survive until hospital discharge than patients who did not. Additionally, patients with longer CPR were less likely to survive up to eight months after the event, which was also influenced by age and length of cardiopulmonary CPR [7].

Cardiac irregularities are more likely to affect men than women, and the risk increases in middle age. The probability of survival up to discharge when a patient is resuscitated in hospital is about 17%. Additional studies are needed to determine the factors associated with the better results and optimal defibrillation time for people with asystole [8,9].

This publication aims to draw the attention of physicians to the problem of asystole occurring after the administration of the described groups of drugs and to increase awareness of the existence of such a problem. Although this study describes cases of episodes of asystole, in some cases cardiac arrest occurred without a decrease in myocardial excitability. It is hoped that there will be more reports of such situations so that drug-induced asystole can be effectively eliminated in the future.

## 2. Drug Categories

The aim of this study was to review the available literature to identify cases of asystole and confirm whether their occurrence was related to the medications taken by the patient. This is an extremely important issue for physicians hoping to eliminate any risk of the condition occurring [10]. Any identified drugs were grouped according to their mechanism of action to simplify the analysis. This study offers a more comprehensive perspective on the various effects, and side effects, of drug treatment and their potential causes. All described patient cases are presented in Table 1.

### 2.1. Calcium L-Channel Blockers

Verapamil and diltiazem are non-dihydropyridine (cardioselective) L-channel blockers, which act by blocking calcium influx inside cardiac cells. These drugs act on the cardiomyocytes to cause reflexive bradycardia and block the asystole. Verapamil is very effective in treating hypertrophic cardiomyopathy in propranolol-ineffective therapy or ventricular tachycardia [31,32]. Diltiazem is mostly used in therapy of vasospastic angina [33]. In addition to asystole and cardiac symptoms, verapamil overdose also results in hypotension, bradycardia and dysrhythmia [34].

Calcium channel blockers prevent cardiac shrinkage by interacting with receptors on the cell membrane of cardiac cells. In cardiac and smooth muscle cells, the influx of extracellular calcium through these channels results in the release of stored calcium from the sarcoplasmic reticulum, resulting in an increase in intracellular calcium. This increase is needed for the production of excitation contraction coupling and muscle contraction. Cardiac and smooth muscle cells have little sarcoplasmic reticulum and are therefore highly dependent on trans-membrane calcium influx for the initiation of intracellular events. This makes cardiac and smooth muscle cells particularly susceptible to calcium channel blockage, which results in reduced myocardial contractility and vasodilatation [35]. These drugs typically have a negative inotropic effect. Verapamil can decrease myocardial contractility. Some reports show that the results vary and may be dependent on doses [32].

Both verapamil and diltiazem overdose result in a decrease in contractile heart function. Overdose results in various symptoms which can lead to cardiac death or pulmonary oedema in critical cases, and sudden death. Sudden death can occur due to excessive vasodilation, sinus node arrest, which is closely connected with the appearance of asystole and atrioventricular blockage resulting from sinus node arrest. This causes hypotension and increased obstruction, thus resulting in a vicious circle: hypotension increases coronary perfusion pressure, which increases left ventricular pressure. These symptoms typically result in asystole or pulmonary oedema in patients with hypertonic cardiomyopathy; this condition is relieved by verapamil [32].

This model of asystole or cardiac arrest is not only theoretical [32]. The number of calcium channel blockers is small, and diltiazem may be used in coronary spasm [36]. In such cases, asystole typically results from drug overdose and its use in combination with b-blockers, especially propranolol, and selective serotonin re-uptake inhibitors (SSRIs). Combining verapamil with propranolol results in severe AV blockage, asystole and cardiogenic shock. While animal studies and some human cases indicate that L-channel blockers can protect cerebral cells from degeneration, verapamil-induced cardiac arrest leads to renal and cardiac degradation [36,37].

Typically, if a drug has been applied, it is important to observe the patient and to increase the dosage only if adverse effects do not occur [32]. While asystole occurs very rarely after overdosing, other side effects can occur, such as varying to severe hypotensive episodes, sinus arrest (which can lead to cardiac arrest), heart blocks, hypotension resulting in fatal cardiac arrest, bradycardia up to severe AV blockage, hyperglycemia and metabolic acidosis [31].

Evans describes the case of a 15-year-old girl who survived a cardiac arrest for a total of 65 min. The patient received 7.2 g verapamil and 240 mg of paroxetine (SSRI) before she was transferred to the hospital. Later tests confirmed a lack of neurological damage, suggesting that verapamil may have neuroprotective properties [36].

Another case concerned the death of an 82-year-old woman who suffered from paroxysmal or chronic atrial fibrillation (AF). The patient suffered cardiac arrest after administration of verapamil and bisoprolol (β-blocker) eight hours previously. The asystole was reported in sinus rhythm or was connected with atrioventricular block during atrial fibrillation. It was suggested that verapamil can raise beta-blocker concentrations by inhibiting their metabolism. Any information about sick sinus syndrome or whether the arrest manifested itself in the restoration of sinus rhythm is absent [38].

The study analyzed the effect of slow intravenous injections of verapamil 7 mg. A 57-year-old man with a history of episodes of supraventricular tachycardia presented to the emergency department. Before admission to the hospital, 600 mg of practolol was administered. Then, verapamil was administered at a dose of 1 mg per minute under ECG monitoring at the hospital. After administering 7 mg of this drug, the patient felt weak and his systolic blood pressure dropped to 70 mm Hg. One minute later, asystole occurred. After rescue, his blood pressure reached 120/80 mm Hg. One explanation for the occurrence of asystole was the administration of a combination of verapamil and practolol. However, after practolol was administered, emergency room personnel did not observe a reduction in heart rate. It was only after the verapamil injection that asystole occurred [11].

Another study examined the impact of verapamil intoxication in rats with asystole. The study group received 16, 100 or 1000 mg/kg of sugammadex infused in 12 mL/kg normal saline for five minutes, while the controls received 12.4 mL/kg normal saline for five minutes. It was found that, while 16 mg/kg sugammadex can delay cardiotoxicity, a 1000 mg/kg dose had the opposite effect [39].

### 2.2. Beta-Blockers

The first β-adrenergic antagonist, pronethalol, was synthesized by Black in 1958 based on Alquist’s postulates. Today, there are nineteen β-adrenergic antagonists [23,30].

Beta-adrenolytic antagonists are mostly used to treat hypertension, angina, tachydysrhythmias, tremor, migraine and panic attacks, and are divided into three generations. Based on the type of beta receptor that they bind to, beta-blockers have different pharmacological properties [40].

Voltage-sensitive calcium channels liberate calcium. Phosphorylation of the L-type calcium channel increases the influx of calcium during each cell depolarization; this increases contractility by triggering a greater release of calcium from the sarcoplasmic reticulum. Cardiac β2-adrenergic receptors are associated with both excitatory Gs proteins and inhibitory Gi proteins [30].

After beta-blocker therapy, the patient should be monitored by physicians in case of down-regulation [41,42]. In particular, it has been found that the use of alpha- or beta dual receptor blockers is associated with a high probability of asystole; this may be due to changes in blood flow influenced by alpha-receptor activity [43,44].

Most of the toxic properties of β-adrenergic antagonists result from their ability to competitively antagonize the action of catecholamines, which stimulate beta- receptors in the heart. In addition, beta-antagonists disturb calcium handling in the sarcoplasmic reticulum and calcium uptake from intracellular organelles. Taken together, these can result in refractory bradycardia [40,45,46].

The administration of propranolol and verapamil has been found to be associated with the duration of potentiation or the length of asystole. Hence, their combined use can increase the strength of asystole. However, propranolol can also induce asystole, even if it is not administered with an acetylcholinesterase inhibitor or another asystole-creating drug because beta-blockers initiate bradycardia and, in overdosage, asystole is possible. In addition to combined verapamil and propranolol, asystole can also be achieved by other combinations of cardioselective calcium channel blockers and beta-blockers; it hence appears that any drug which stimulates the vagal nerve has a definite chance of inducing asystole [47].

In many cases, the use of any-generation beta blockers and non-dihydropyridine calcium channel antagonists may result in asystole, extreme bradycardia, complete atrioventricular blockage and may depress ventricular contraction. These two drugs together should be used very rarely, especially if the patient does not use a pacemaker and control of ventricular rate control is indicated [48]. Prolonged QRS and QT intervals can occur, and severe poisonings result in cardiac arrest [13,49]. In cases when patients have cardiac or renal damage, labetalol administration and low halothane concentration can lead to cardiac death [50]. Also, there is no evidence that β-blockers improve prognosis in patients with heart failure and AF [49].

β-adrenergic antagonist overdose typically results in bradycardia and hypotension, with asystole observed in critical cases. Beta-blocker overdose is best treated with glucagon, although high doses of insulin with glucose presents a promising new treatment. Catecholamine infusions can be helpful but should be closely monitored by hospital staff. Patients who fail this treatment may respond to intravenous fat emulsion therapy, phosphodiesterase inhibitors or mechanical support of circulation. Fortunately, most patients do not need aggressive types of therapy [13,40,51].

#### 2.2.1. Labetalol

In one case, a 48-year-old woman had a cardiac arrest during surgical treatment of phaeochromocytoma. Before the operation, the patient had received labetalol to blockade the alpha receptors; however, she experienced asystole during the operation. The most likely cause of cardiac arrest was the combination of vagal nerve stimulation by laryngoscopy and the alpha-receptor blocker [19].

Another study described another case of a 49-year-old woman who experienced asystole during an operation. Resuscitation was attempted using 1.8 mg atropine, 1 mL of 1 in 1000 adrenaline and 10 mL of 10% calcium gluconate solution, intravenous infusion of 2 mg isoprenaline. The patient was receiving labetalol for hypertension and cardiac problems. Unfortunately, the patient died during reanimation. This case suggests that the combination of labetalol and low-concentration halothane can result in sudden cardiac death in the event of myocardial damage [50].

#### 2.2.2. Others β-Adrenergic Antagonist

One case of death due to asystole following an overdose of alprenolol in a 32-year-old woman has been reported. There is also a known case of oxprenolol overdose in a 57-year-old woman. This led to coma and eventually death from asystole. A 24-year-old woman and in a 41-year-old man developed asystole following an overdose of propranolol combined with alcohol [52].

### 2.3. Potassium Channel Blockers

The potassium channel blockers comprise drugs which block potassium channel without any blockage of the sodium channel, and those with beta- antagonistic activity. They are typically used to treat arrhythmia and create a third group antiarrhythmic drugs, by stopping the potassium current in the cell. The indications for the use of these drugs have been well known for many years. Briefly, the mode of potassium channel blockers action is based on prolonged repolarization without any influence of conduction velocity. Antiarrhythmic drugs selectively prolong cardiac action potential by blocking potassium channels in effect potassium currents in cardiac cells, and lengthen the effective refractory period without affecting the maximal rising rate of phase 0. The best-known chemical substances from this group are sotalol, a beta-blocker with a dual mode of action, and amiodarone. Sotalol is a non-cardioselective P-adrenoceptor blocker that acts on the potassium channel; it achieves its inotropic effect by not depressing cardiac contractility. Another well-known antiarrhythmic drug is amiodarone, which acts by lengthening the action potential and inhibiting the maximal rising rate of depolarization [53,54].

Sotalol has the same mode of activity as other beta-blockers; it also prolongs the action potential and refractory period characteristic for the class 3 antiarrhythmic medicines. The characteristic symptoms of sotalol poisoning include prolonged Q-T interval, multifocal ventricular extrasystoles, ventricular tachycardia and asystole, which is characteristic of beta-blockers. While cases of fatal sotalol overdose have been reported at doses of 3.2 g and 14.4 g, recovery has been noted after a 16 g dose [18,55]. Sotalol can induce asystole because of its membrane-stabilizing properties, which are exacerbated by its beta-blocker properties. Toxic effects are observed above a concentration of 5.1 pg/mL [18,56].

Amiodarone is believed to induce asystole by three routes: causing sinus arrest or enhancing suppression of sinus node, decreasing the automaticity of an ectopic atrial pacemaker or by causing an exit blockage from the site of the pacemaker and suppressing subsidiary escape pacemakers. Experiments on rabbits indicate that amiodarone decreases the SA node. The side effects of amiodarone treatment include cardiac arrest, sinus bradycardia and prolonging SA node recovery time and SA conduction [57].

#### 2.3.1. Sotalol

Like all beta- blockers, sotalol can cause asystole. One characteristic feature of sotalol is its ability to prolong QT. A 70-year-old woman attempted suicide by taking 6.72 g sotalol about 12 h earlier. When the patient was admitted to hospital, the healthcare team administered activated charcoal and glucagon and, two hours later, the heart rate decreased from 50 to 25 beats per minute. It was decided to administer atropine sulfate (0.5 mg) intravenously but it was ineffective. The next step was to administer an infusion of isoprenaline (10 μg/min) to the patient. Suddenly, two asystole episodes occurred. External chest compression and adrenaline administration was started. When the situation was controlled, dopamine was temporarily administered for low blood pressure, and the glucagon and isoprenaline infusions were withdrawn. No further renal or heart complications were noted [55].

Another case concerned a 58-year-old woman who died due to cardiac arrest due to sotalol. The woman had attempted to commit suicide by taking 14.4 g of sotalol (90 tablets of 160 mg each) and 50 mg of triazolam. The patient was admitted to hospital, but died 3.5 h later. The autopsy showed high sotalol concentration in all tissues of the body. The highest sotalol concentration was in the muscle, lung and heart [56].

#### 2.3.2. Amiodaron

A 27-year-old Caucasian woman was treated for persistent atrial tachycardia. After the treatment failed, she was administered digoxin 0.25 mg, nadolol 80 mg and amiodarone 1 mg per day. After five days, the patient complained of recurrent episodes of presyncope. Electrocardiogram showed repeated episodes of asystole, with the durations of three reported episodes being about 5, 6 and 9 s. The level of amiodarone in serum was 1.1 g/mL. She was prescribed digoxin (0.25 mg/day), amiodarone (400 mg/day) and nadolol (40 mg/day), and a permanent transvenous VVI pacemaker was implanted. Although the patient received three different drugs, amiodarone was found to be the source of the asystole episodes [57].

### 2.4. Drugs Used in Anesthesia, Sedation and Premedication

#### 2.4.1. Propofol

Propofol is a phenyl chemical compound used as anesthetic agent; however, it appears to cause more bradycardia and asystole episodes than another anesthetic. This relationship between cardiac arrest and propofol administration has been noted in many studies. Tramèr examined the relationship between propofol use and bradycardia and asystole based on data from 1987 to 1995; it was found that asystole was reported 13 times in 17 reports from 10 countries, with one death. These findings underline the care needed by physicians working with propofol anesthesia. Data from twelve drug monitoring centers reported 65 asystole episodes, of which 14 were fatal. However, the full analysis found fewer than 35 episodes in 10,000 patients, and less 12 deaths caused by bradycardia out of 100,000 cases. The longest episode of asystole in surviving patients was 45 s. Propofol overdose can cause respiratory depression and consequent cardiac arrest [28].

One case described a 26-year-old woman who received laparoscopic tubal ligation as a one-day procedure. Preliminary interviews did not reveal any cardiac, respiratory, renal or endocrine symptomatology. The patient had never fainted. After preoperative preparation, the patient received fentanyl and bolus of propofol. A few seconds after the propofol bolus, the ECG changed suddenly from sinus rhythm. The length of asystole suggested that propofol induced cardiac arrest. Based on literature reports, the authors observed that the onset of asystole during anesthesia induction was often consistent with a period of extreme hyperexcitability of the vagus nerve. The data also indicated that the event was likely caused by propofol [24].

Propofol infusion syndrome (PRIS) is a rare but grave complication, which can be observed after propofol infusion. Two of the most serious symptoms are cardiac arrest and asystole. The main symptom of myocardial infarction, but also the result, is cardiovascular dysfunction, with particular emphasis on impaired contractility of the cardiovascular system, metabolic acidosis, lactate acidosis, rhabdomyolysis, hyperkalemia, lipidemia, liver enlargement, acute renal failure and in most cases mortality. Symptoms that may occur on the cardiovascular side include right bundle branch block, hypotension, brugada-like syndrome ECG presentation (ST-segment elevation and widening of the QRS complex), ventricular tachycardia, ventricular arrhythmia, supraventricular tachycardia, atrial fibrillation, cardiogenic shock and asystole. Propofol, on the other hand, is mainly used to induce anesthesia, but adverse cardiovascular effects were observed in a 20-year-old woman after a propofol infusion treated for the onset of epileptic symptoms [15,58].

A 42-year-old woman suffering from severe rheumatoid arthritis underwent surgery. The patient took her usual medications on the day of surgery. At the beginning of the operation, she received fentanyl, propofol and succiniyholine. After 20–30 s from the succinylcholine bolus, the sinus rhythm changed abruptly to asystole. Atropine was administered, and heart rate increased to 80 beats per minute. In this clinical example, asystole occurred after taking many medications, and it is very difficult to say which one of them is the cause. A number of medications used for induction of anesthesia have been found to induce severe bradycardia followed by asystole [14].

#### 2.4.2. Dexmedetomidine

Dexmedetomidine, with its sedative and anti-anxiety properties, is indicated for patients requiring sedation and is suitable for premedication and analgesia. Patients are more awake and pain-free when dexmedetomidine is used than with infusions of propofol and fentanyl. Because of its high price, it is more often used in combination with lower doses of propofol and fentanyl and to wean patients from mechanical ventilation. Both clinical pharmacists and physicians should ensure that the correct dose is administered to prevent adverse effects such as bradycardia and asystole, or the cumulative effects of dexmedetomidine in patients with multiple organ failure especially in the intensive care unit. Cases of asystole after dexmedetomidine infusion during sedation for mechanical ventilation in patients with acute respiratory distress syndrome (ARDS) were reported by Noelle et al. In all cases described, patients were treated in the intensive care unit [12,59].

A 67-year-old male with schizophrenia, hepatitis C, hypertension and hyperlipidemia was admitted to accident and emergency after a fall. The medical staff suspected a stroke. After an initial evaluation, the patient was intubated and kept on mechanical ventilation. The patient received an infusion of propofol and dexmedetomidine for sedation. During the SBT (spontaneous breathing trial), the patient began to cough and his heart rate immediately dropped, which was followed by a one-minute asystole before the cardiac muscle spontaneously returned to normal rhythm. Fifteen minutes later, another asystole episode occurred, which again spontaneously resolved. At this time, the patient was given atropine, dexmedetomidine was discontinued and propofol was restarted [12].

A 49-year-old man suffering from epilepsy was admitted to accident and emergency with acute hypoxic respiratory failure caused by SARS CoV-2 pneumonia. After 11 days of hospitalization, dexmedetomidine was administered as a sedative and analgesic during mechanical intubation. Some days later, the patient started coughing, and severe sinus bradycardia occurred. This was followed by an episode of asystole, which lasted no longer than 10 s. The patient’s heart rate returned to normal without any intervention after dexmedetomidine was stopped. The authors believe that dexmedetomidine infusion was the main cause of asystole [12].

A 58-year-old man suffering from SARS-CoV-2 was admitted to the hospital. He complained of fever, shortness of breath and fatigue for two weeks prior to admission. He had a rich disease history including hepatitis C, hypertension and hyperlipidemia. Dexmedetomidine was administered from the first day of admission during mechanical ventilation. After three weeks of hospitalization, two 10 s episodes of asystole were noted. Following this, dexmedetomidine was stopped. The authors attribute the asystole to the combination of vagal responses associated with the severe cough and dexmedetomidine infusion [12].

A 57-year-old man suffering from chronic obstructive pulmonary disease (COPD), hyperlipidemia, hypertension and bilateral carotid stents was admitted for elective aortobilateral profunda bypasses. During mechanical intubation, propofol, fentanyl and dexmedetomidine were administered for sedation. The next day and the day after, a ten-seconds asystole occurred [12].

A 73-year-old male patient without any history of cardio-vascular diseases received a transurethral lithotomy for bilateral renal stones. During general anesthesia used for the surgery, asystole occurred as a complication of dexmedetomidine, spinal anesthesia and vagal nerve reflex. Dexmedetomidine induces bradycardia in 9–42% patients receiving mechanical ventilation [16].

### 2.5. Other Drugs

Asystole has also been associated with a small number of other drugs; in particular, pyridostigmine, hydrocodone and acetaminophen. As such, appropriate care should be taken when using them.

#### 2.5.1. Adenosine

Adenosine is an organic chemical substance built of ribose and adenine. The compound has a short-term action on the cardiac muscle and expands the blood vessels. Due to its short life in the circulation, adenosine is used in the treatment of paroxysmal supraventricular tachycardia, as well as in radionuclide myocardial perfusion imaging and digital three-dimensional reconstruction [20,60].

The inhibition of conduction time is due to its effect on receptors in the AV node of the heart. Adenosine activates specific potassium channels, leading to the removal of potassium outside the cell membrane. This action results in hyperpolarization of the resting potential. Concomitant arrest of calcium ion flow leads to inhibition of calcium-dependent action potentials, ultimately disrupting the resting membrane potential of the slow cardiac nodal myocyte. All actions result in inhibition of conduction in cardiac cells and cause asystole [29,61].

The most common side effects of adenosine use include headache, hypotension, shortness of breath, tightness of the chest, metallic taste, tightness in the throat and pressure in the groin [62,63].

Adenosine is used in intracranial aneurysm surgery, because it induces asystole, and can reduce bleeding while removing aneurysms. A review of all medical reports of patients undergoing intracranial aneurysm surgery between July 2016 and December 2018 identified a relationship between adenosine dosage and the frequency of occurrence of asystole. In this time period, nine intracranial aneurysm operations were performed for eight patients. Thirteen asystole cases were reported, five to reduce bleeding. The doses were 9 mg (0.20 mg/kg), 12 mg (0.25 mg/kg), 12 mg (0.26 mg/kg), 18 mg (0.34 mg/kg) and 18 mg (0.39 mg/kg). The cardiac arrests lasted 12, 14, 9, 44 and 18 s. Without intraoperative aneurysm rupture, asystole occurred after the application of 9–18 mg doses and the cardiac arrest lasted from 9 to 33 s. The authors report that a low dose of adenosine (0.2–0.4 mg/kg) is safe in surgical operations, while an initial dosage of 6 mg or 0.1–0.2 mg/kg can induce short-term asystole [64].

Another study examined the effect of per-procedural digital three-dimensional reconstruction 3DRA of the left atrium, right atrium, left ventricle, right ventricular group in 40 patients aged 55 to 67 years. None of the patients had asthma, symptoms of angina or congestive heart failure, nor therapy with dipyridamole or theophylline. In 39 cases, adenosine application yielded positive effects which could be seen in the connection between higher 3D image qualities with a lower contrast. The results indicate that adenosine doses between 60 and 100 mg are more effective than 50 mg, and that adenosine induces asystole. This feature is highly desirable in 3DRA, because view is clear and transparent [20].

Studies by the Biomedical Research Institute, Harbor-UCLA Medical Center examined the induction of asystole by adenosine. Adenosine inhibits sinus node automaticity, thus suppressing atrioventricular nodal conduction, resulting in atrioventricular (AV) blockage and finally asystole. All patients who received adenosine intravenously at different doses experienced asystole episodes. These findings emphasize the importance of understanding the possible effects of adenosine treatment, especially the predictable side effects [65].

#### 2.5.2. Ceftriaxone

Ceftriaxone is an organic chemical compound, a third-generation cephalosporin that can inhibit bacteria resistant to classic β-lactamases. While the most common side effect is a skin reaction, asystole has been reported after even a single dose. In one case, a 55-year-old man was admitted to the emergency department with high fever, stomach pain, dysuria and weakness. After initial examination, he was administered ceftriaxone as an infusion, resulting in asystole after one minute; however, circulation was restored after 20 min [66].

#### 2.5.3. Hydrocodone or Acetaminophen

In another case, a 56-year-old woman reported to hospital with weakness, dizziness, nausea and a near-syncope condition, together with leg and plantar foot pain. No hypertension, peripheral vascular disease or cerebrovascular events were noted. The patient reported that she had taken a combination of hydrocodone and acetaminophen the night before. While the patient was in the emergency department, she experienced a few asystole episodes lasting 6 to 8 s, each of which resolved to a normal sinus rhythm. In this case, asystole occurred as an effect of an extreme vasovagal response. All other causes of asystole were ruled out [21].

#### 2.5.4. Lansoprazole

Lansoprazole is a proton pump inhibitor (PPI) commonly prescribed for stomach complaints. However, a case of asystole has been reported after the first dose of lansoprazole. A 43-year-old woman with diabetes and a coronary angiography due to dyspeptic symptoms lost consciousness within minutes of taking 30 mg of lansoprazole. The patient was intubated, and symptoms of asystole were observed after the administration of lansoprazole. After admission to the hospital, the first rhythm analyzed by doctors was asystole. Additionally, the patient was given 0.5 mg of epinephrine and 2 mg per kg of methylprednisolone. Unfortunately, long-term hypoxia caused multiorgan dysfunction syndrome and the patient could not be saved. There are known cases of anaphylaxis after taking lansoprazole in the literature. One of the rarest consequences of anaphylactic shock is bradycardia with hypotension, which in extreme cases can lead to asystole. However, this is the only case in which respiratory arrest and ultimately asystole occurred. A second likely cause of asystole is the negative effect on reduced cardiac contractility. Lansoprazole administered intravenously and orally causes cardiac deceleration, ventricular arrhythmias and cardiac arrest. The mechanism of action includes decreased cardiac contractility at higher concentrations, while reducing Ca^2+^ signaling and myofilament activity, which can ultimately lead to asystole [17,22,27].

#### 2.5.5. Neostigmine/Glycopyrrolate

Neostigmine is commonly used to reverse the action of non-depolarizing muscle relaxants. In one case, a 68-year-old man with end-stage renal disease underwent laparoscopic insertion of a peritoneal dialysis catheter and umbilical hernia repair. At the end of the procedure, a combination of neostigmine/glycopyrrolate was used to reverse neuromuscular blockage; however, the patient experienced three episodes of asystole. The first occurred three hours after neostigmine/glycopyrrolate administration and lasted 5 s, the second occurred after five hours and lasted 15 s, and the last one after 11 h, and lasted one minute (requiring chest compressions). It should be noted that general anesthesia was induced with propofol, but asystole was noted only after the administration of neostigmine. The patient was discharged and no more recurrences of asystole were found [26].

#### 2.5.6. Pyridostigmine

A 68-year-old woman was admitted to hospital requiring oral pyridostigmine administration and intravenous (IV) methylprednisolone. On the second day, bradycardia occurred, with a heart rate ranging from 40 to 50/min and frequent sinus pauses lasting from 1 to 3 s. This was followed by asystole lasting 16 s. After the bradycardia and asystole, pyridostygmine was replaced with hyoscyamine, resulting in an improvement in bradycardia. The patient had no previous history of bradyarrhythmias or dysautonomia. Clearly, the risk of bradycardia or asystole should be considered during treatment with pyridostigmine [25].

#### 2.5.7. Regadenoson

A 58-year-old man presented with noticeable dyspnea after walking. It was decided to obtain a resting perfusion image with ^99m^Tc tetrofosomine. Approximately one minute after initiating 0.4 mg regadenoson, i.e., the standard dose, and intravenous ^99m^Tc tetrofosmin, the patient felt faint, lost consciousness and developed asystole. Fortunately, after a minute of chest compressions and aminophylline treatment, the patient regained consciousness. The patient was admitted to hospital for observation and then discharged without a repeat episode. It is possible that the symptoms may have occurred in response to a drug interaction as the patient was taking n-acetylcysteine. Clearly, further data are needed on the safety of regadenoson in patients with pulmonary fibrosis taking n-acetylcysteine [67].

### 2.6. Article Selection Process and Selection Criteria

The total number of articles from Pubmed was 1951. A total of 76 articles were obtained from Web of Science and 352 from Scopus. From the 2379 articles, a final number of 2134 articles were excluded, based on eligibility criteria. Articles that were not in English were not considered for the review. Articles that were on general aspects associated with cardiac arrest and not pertaining to the subject were not considered. Articles that were related to asystole and some drug administrations were considered. A final number of 71 articles were included for the review.

## 3. Discussion

**Figure 1:**
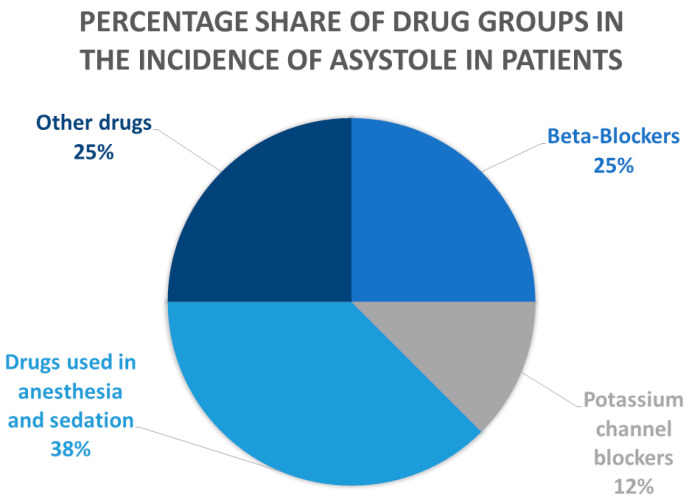
Percentage share of drug groups in the incidence of asystole in patient described in the publication.

While the precise etiopathogenesis of asystole is unclear, it is known to be influenced by the cause and the timing of the medical intervention. However, its rapidity makes it difficult to stabilize, and the prognosis is generally poor [68]. Some models of asystole may be caused by excessive administration of calcium L-channel blockers such as verapamil and diltiazem, which are used for treating conditions associated with cardiological problems [33,34]. More often, however, asystole has been found to result from the combination of these drugs with beta-blockers, particularly second and third-generation drugs: it is believed that verapamil inhibits their metabolism, resulting in their accumulation in the body, and increasing the chance of patient death [37,40]. The most dangerous beta-blockers include propranolol, whose overdose is closely related to the occurrence of asystole [52]. The application of propranolol and verapamil has been shown to affect the duration and strength of asystole [48]. Although verapamil has been found to have superior neuroprotective ability, some studies have found diltiazem to be more effective in treating cardiovascular diseases [34,37]. The incidence of asystole depending on the drug taken from a specific group is presented in Figure 1.

Another group of drugs meriting attention are the potassium channel blockers. Combining sotalol with beta-blockers contributes to the development of asystole [57]. Asystole has also been associated with the use of other drugs such as amiodarone, the anesthetic propofol [55] and various angiotensin-converting enzyme (ACE) drugs used to lower blood and reduce the risk of heart failure [13].

In most published cases, the main cause of drug initiation of asystole was associated with overdose or an inappropriate combination of drugs. There is hence great interest in identifying drugs that could reduce the likelihood of cardiac arrest, such as captopril and enalapril [20,60]. Also, pacemakers have been found to be potential life savers in patients prone to cardiac arrest [10].

Asystole is characterized by a pattern: bradycardia, followed by cardiac arrest, which in most cases eventually leads precisely to asystole. Thus, this study shows the possibility of cardiac arrest with cardiac excitability progressing to asystole.

Our review suggests that, while asystole may affect younger and older patients, it is more common in the latter. This may be due to the nature of the diseases that require drugs that can potentially cause asystole. There is a need for greater reporting of cases by physicians to identify pharmacological compounds that may cause asystole in patients. In the meantime, only rapid application of resuscitation measures and medical interventions can reduce mortality rates in such cases.

## 4. Conclusions

There are several main groups of drugs (beta-blockers, potassium channel blockers and drugs used in anesthesia and sedation), and several individual drugs from other groups, when taken, may result in patients suffering from asystole. In these cases, physicians should pay special attention when prescribing them or, if possible, change the therapy to another drug. Unfortunately, not all cases of asystole are published, which causes a lack of knowledge. Awareness of the problem of asystole is increasing and there is a possibility of preventing asystole caused by giving patients medication in the near future.

## Figures and Tables

**Table 1:** Cases of asystole and cardiac arrest in patients after taking certain medications. (ND—no data).

Age (Years)	Sex	Health Problem	Treatment	Drug Induce Asystole	Reference
15	female	drug overdose	verapamil and paroxetine	verapamil	[11]
20	female	observation after childbirth	propofol	propofol	[12]
24	female	drug overdose	propranolol	propranolol	[13]
26	female	laparoscopic tubal ligation	fentanyl and propofol	propofol	[14]
27	female	persistent atrial tachycardia	digoxin, nadolol and amiodarone	amiodarone	[15]
32	female	drug overdose	alprenolol	alprenolol	[13]
41	male	drug overdose with alcohol	propranolol	propranolol	[13]
42	female	severe rheumatoid arthritis	midazolam, fentanyl, pancuronium, propofol and succinylcholine	propofol succinylcholine	[16]
43	female	diabetes, coronary angiography	lansoprazole	lansoprazole	[17]
48	female	surgery	labetalol	labetalol	[18]
49	female	surgery	labetalol	labetalol	[19]
49	male	acute hypoxic respiratory failure caused by SARS CoV-2	dexmedetomidine	dexmedetomidine	[20]
55	male	high fever, stomach pain, dysuria and weakness	ceftriaxone	ceftriaxone	[21]
56	female	weakness, intermittent nausea, dizziness, and near-syncope	hydrocodone and acetaminophen	hydrocodone/acetaminophen	[22]
57	female	drug overdose	oxprenolol	oxprenolol	[13]
57	male	chronic obstructive pulmonary disease, hypertension, hyperlipidemia and bilateral carotid	propofol, fentanyl and dexmedetomidine	dexmedetomidine	[20]
57	male	supraventricular tachycardia	vigorous thumps on the chest	Verapamil and practolol	[23]
58	female	drug overdose	sotalol and tiazolam	sotalol	[24]
58	male	SARS CoV-2	dexmedetomidine	dexmedetomidine	[20]
58	male	dyspnea	tetrofosomine, regadenoson	redagenoson	[25]
63	male	diabetes mellitus, chronic obstructive pulmonary disease, hypertension, hyperlipidemia and tobacco abuse	dexmedetomidine	dexmedetomidine	[20]
65	female	diplopia, dysphagia and muscle weakness, myasthenia gravis	pyridostigmine	pyridostigmine	[26]
67	male	stroke	propofol and dexmedetomidine	dexmedetomidine	[20]
68	male	laparoscopic insertion of a peritoneal dialysis catheter and umbilical hernia repair	neostigmine and glycopyrrolate	neostigmine/glycopyrrolate	[27]
					
70	female	drug overdose	Mianserin hydrochloride and sulpiride	sotalol	[28]
73	male	bilateral renal stones	dexmedetomidine	dexmedetomidine	[29]
82	female	congestive heart failure	verapamil and bisoprolol	verapamil	[30]

## Data Availability

All data generated or analyzed during this study are included in this published article.

## References

[1] Rocamora R., Kurthen M., Lickfett L., Von Oertzen J., Elger C.E. (2003). Cardiac asystole in epilepsy: Clinical and neurophysiologic features. Epilepsia.

[2] Fontenla A., Miguel A., López-Gil M., Arribas F. (2014). An unusual mechanism of asystole. J. Electrocardiol..

[3] Brancheau D., Blanco J., Gholkar G., Patel B., Machado C. (2016). Cannabis induced asystole. J. Electrocardiol..

[4] Heckle M.R., Nayyar M., Sinclair S.E., Weber K.T. (2018). Cannabinoids and Symptomatic Bradycardia. Am. J. Med. Sci..

[5] Li W., Jayagopal L.A., Taraschenko O. (2019). Ictal asystole with isolated syncope: A case report and literature review. Epilepsy Behav. Case Rep..

[6] Myerburg R.J., Halperin H., Egan D.A., Boineau R., Chugh S.S., Gillis A.M., Goldhaber J.I., Lathrop D.A., Liu P., Niemann J.T. (2013). Pulseless electric activity: Definition, causes, mechanisms, management, and research priorities for the next decade: Report from a national heart, lung, and blood institute workshop. Circulation.

[7] Kutsogiannis D.J., Bagshaw S.M., Laing B., Brindley P.G. (2011). Predictors of survival after cardiac or respiratory arrest in critical care units. CMAJ. Can. Med. Assoc. J..

[8] Cheema M.A., Ullah W., Abdullah H.M.A., Haq S., Ahmad A., Balaratna A. (2019). Duration of in-hospital cardiopulmonary resuscitation and its effect on survival. Indian. Heart J..

[9] Kajino K., Iwami T., Daya M., Nishiuchi T., Hayashi Y., Ikeuchi H., Tanaka H., Shimazu T., Sugimoto H. (2008). Subsequent ventricular fibrillation and survival in out-of-hospital cardiac arrests presenting with PEA or asystole. Resuscitation.

[10] Zanoni S., Platt G., Carstairs S., Hernandez M. (2014). Complete ventricular asystole in a patient with altered mental status. West. J. Emerg. Med..

[11] Krikler D., Spurrell R. (1972). Asystole after Verapamil. Br. Med. J..

[12] Noelle P., Olivia J., Aaron B., Jaber M.-H. (2022). Transient Asystole Linked to Dexmedetomidine Infusion. Int. J. Crit. Care Emerg. Med..

[13] Rathore K.S., Nema R.K., Sisodia S.S. (2010). Timolol maleate a gold standard drug in glaucoma used as ocular films and inserts: An overview. Int. J. Pharm. Sci. Rev. Res..

[14] Egan T.D., Brock-Utne J.G. (1991). Asystole after anesthesia induction with a fentanyl, propofol, and succinylcholine sequence. Anesth. Analg..

[15] Mallow Corbett S., Montoya I.D., Moore F.A. (2008). Propofol-related infusion syndrome in intensive care patients. Pharmacotherapy.

[16] Amano E., Tateda T., Inoue S. (2018). Asystole During Administration of Dexmedetomidine with Spinal Anesthesia: A Case Report. J. St. Marian. Univ..

[17] Candar M., Gunes H., Boz B.V., Kandis H., Kutlucan L., Saritas A. (2014). Asystole after the first dose of lansoprazole. Am. J. Emerg. Med..

[18] Montagna M., Groppi A. (1980). Fatal sotalol poisoning. Arch. Toxicol..

[19] Taylor M.J., McIndoe A. (2007). Unresponsive asystolic cardiac arrest responding to external cardiac pacing in a patient with phaeochromocytoma. Anaesthesia.

[20] Ector J., De Buck S., Nuyens D., Rossenbacker T., Huybrechts W., Gopal R., Maes F., Heidbüchel H. (2009). Adenosine-induced ventricular asystole or rapid ventricular pacing to enhance three-dimensional rotational imaging during cardiac ablation procedures. Europace.

[21] Sudhakaran S., Surani S.S., Surani S.R. (2014). Prolonged ventricular asystole: A rare adverse effect of hydrocodone use. Am. J. Case Rep..

[22] Mitsuboshi S., Imai S., Kizaki H., Hori S. (2024). Concomitant use of lansoprazole and ceftriaxone is associated with an increased risk of ventricular arrhythmias and cardiac arrest in a large Japanese hospital database. J. Infect..

[23] Stapleton M.P. (1997). Sir James black and propranolol the role of the basic sciences in the history of cardiovascular pharmacology. Tex. Heart Inst. J..

[24] Guise P.A. (1991). Asystole following propofol and fentanyl in an anxious patient. Anaesth. Intensive Care.

[25] Khan M.S., Tiwari A., Khan Z., Sharma H., Taleb M., Hammersley J. (2017). Pyridostigmine Induced Prolonged Asystole in a Patient with Myasthenia Gravis Successfully Treated with Hyoscyamine. Case Rep. Cardiol..

[26] Cachemaille M., Olofsson M., Livio F., Pascale P., Zingg T., Boegli Y. (2017). Recurrent Asystole After Neostigmine in a Heart Transplant Recipient With End-Stage Renal Disease. J. Cardiothorac. Vasc. Anesth..

[27] Tanaka S., Nishigaki K., Ojio S., Okubo M., Yasuda S., Ishihara Y., Kubota T., Takasugi N., Kawamura I., Yamaki T. (2008). Can negative cardiac effect of proton pump inhibitor and high-dose H2-blocker have clinical influence on patients with stable angina?. J. Cardiol..

[28] Tramèr M.R., Moore R.A., Mcquay H.J. (1997). Propofol and bradycardia: Causation, frequency and severity. Br. J. Anaesth..

[29] Page R.L., Joglar J.A., Caldwell M.A., Calkins H., Conti J.B., Deal B.J., Estes N.M., Field M.E., Goldberger Z.D., Hammill S.C. (2016). 2015 ACC/AHA/HRS Guideline for the Management of Adult Patients With Supraventricular Tachycardia: A Report of the American College of Cardiology/American Heart Association Task Force on Clinical Practice Guidelines and the Heart Rhythm Society. J. Am. Coll. Cardiol..

[30] Levitzki A., Marbach I., Bar-Sinai A. (1993). The signal transduction between β-receptors and adenylyl cyclase. Life Sci..

[31] Radford D. (1983). Side effects of verapamil in infants. Arch. Dis. Child..

[32] Epstein S.E., Rosing D.R. (1981). Verapamil: Its potential for causing serious complications in patients with hypertrophic cardiomyopathy. Circulation.

[33] Zhang Z.P., Su X., Liu C.W., Peng J., Song D., Liu B., Wu M.-X., Yang Y.-C. (2015). Heart block or cardiac arrest is not a contraindication for intravenous treatment with diltiazem in the setting of coronary spasm. Am. J. Emerg. Med..

[34] Reines E., Johnsen A., Husum D., Jensen G. (1987). Verapamil intoxication. Report of a fatal case and review of the literature. Ugeskr. Laeger.

[35] MacDonald D., Alguire P.C. (1992). Case report: Fatal overdose with sustained-release verapamil. Am. J. Med. Sci..

[36] Evans J.S.M., Oram M.P. (1999). Neurological recovery after prolonged verapamil-induced cardiac arrest. Anaesth. Intensive Care.

[37] Plumb V.J., Karp R.B., Kouchoukos N.T., Zorn G.L., James T.N., Waldo A.L. (1982). Verapamil therapy of atrial fibrillation and atrial flutter following cardiac operation. J. Thorac. Cardiovasc. Surg..

[38] Déniel A., Fedrizzi S., Lelong-Boulouard V., Coquerel A., Alexandre J. (2016). Fatal cardiac arrest associated with concomitant bisoprolol and verapamil overdose. J. Am. Geriatr. Soc..

[39] Ozbilgin S., Ozbilgin M., Kucukoztas B., Kamaci G., Unek T., Yurtlu B.S., Güneli M.E., Hanci V., Gunerli A. (2013). Evaluation of the effectiveness of sugammadex for verapamil intoxication. Basic. Clin. Pharmacol. Toxicol..

[40] Oliver E., Mayor F., D’Ocon P. (2019). Beta-blockers: Historical Perspective and Mechanisms of Action. Rev. Española Cardiol..

[41] Ladage D., Schwinger R.H.G., Brixius K. (2013). Cardio-Selective Beta-Blocker: Pharmacological Evidence and Their Influence on Exercise Capacity. Cardiovasc. Ther..

[42] Ohkuma S., Katsura M., Shibasaki M., Tsujimura A., Hirouchi M. (2006). Expression of β-adrenergic receptor up-regulation is mediated by two different processes. Brain Res..

[43] Youngquist S.T., Kaji A.H., Niemann J.T. (2008). Beta-blocker use and the changing epidemiology of out-of-hospital cardiac arrest rhythms. Resuscitation.

[44] Barcella C.A., Eroglu T.E., Hulleman M., Granfeldt A., Souverein P.C., Mohr G.H., Koster R.W., Wissenberg M., de Boer A., Torp-Pedersen C. (2020). Association of beta-blockers and first-registered heart rhythm in out-of-hospital cardiac arrest: Real-world data from population-based cohorts across two European countries. EP Eur..

[45] De Wildt D., Sangster B., Langemeijer J., Groot G.d. (1984). Different toxicological profiles for various beta-blocking agents on cardiac function in isolated rat hearts. Clin. Toxicol..

[46] Langemeijer J., De Wildt D., de Groot G., Sangster B. (1986). Calcium interferes with the cardiodepressive effects of beta-blocker overdose in isolated rat hearts. Clin. Toxicol..

[47] Bufkin B.L., Puskas J.D., Vinten-Johansen J., Shearer S.T., Guyton R.A. (1998). Controlled intermittent asystole: Pharmacologic potentiation of vagal- induced asystole. Ann. Thorac. Surg..

[48] Kirch W., Kleinbloesem C.H., Belz G.G. (1990). Drug interactions with calcium antagonists. Pharmacol. Ther..

[49] Mareev Y., Cleland J.G.F. (2015). Should β-Blockers Be Used in Patients with Heart Failure and Atrial Fibrillation?. Clin Ther..

[50] (1979). HUNTERJM Synergism between halothane and labetalol. Anaesthesia.

[51] Frishman W.H., Kowalski M., Nagnur S., Warshafsky S., Sica D. (2001). Cardiovascular considerations in using topical, oral, and intravenous drugs for the treatment of glaucoma and ocular hypertension: Focus on β-adrenergic blockade. Heart Disease.

[52] Frishman W., Jacob H., Eisenberg E., Ribner H. (1979). Clinical pharmacology of the new beta-adrenergic blocking drugs. Part 8. Self-poisoning with beta-adrenoceptor blocking agents: Recognition and management. Am. Heart J..

[53] Mortensen E., Yang T., Refsum H. (1993). Potassium Channel Blockade as an Antiarrhythmic Principle. Cardiovasc. Drug Rev..

[54] Singh B.N., Nademanee K. (1985). Control of cardiac arrhythmias by selective lengthening of repolarization: Theoretic considerations and clinical observations. Am. Heart J..

[55] Adlerfliegel F., Leeman M., Demaeyer P., Kahn R.J. (1993). Sotalol poisoning associated with asystole. Intensive Care Med..

[56] Perrot D., Bui-Xuan B., Lang J., Bouffard Y., Delafosse B., Faucon G., Motin J. (1988). A case of sotalol poisoning with fatal outcome. Clin. Toxicol..

[57] Kuo C.S., Pratap Reddy C., Paciotti M.V. (1985). Asystole during treatment with amiodarone in a patient with persistent atrial tachycardia. J. Electrocardiol..

[58] Mayette M., Gonda J., Hsu J.L., Mihm F.G. (2013). Propofol infusion syndrome resuscitation with extracorporeal life support: A case report and review of the literature. Ann. Intensiv. Care.

[59] Scott-Warren V.L., Sebastian J. (2016). Dexmedetomidine: Its use in intensive care medicine and anaesthesia. BJA Educ..

[60] Jacobson K.A. (2009). Introduction to adenosine receptors as therapeutic targets. Handbook of Experimental Pharmacology.

[61] Rankin A.C., Brooks R., Ruskin J.N., McGovern B.A. (1992). Adenosine and the treatment of supraventricular tachycardia. Am. J. Med..

[62] Luostarinen T., Takala R.S.K., Niemi T.T., Katila A.J., Niemelä M., Hernesniemi J., Randell T. (2010). Adenosine-induced cardiac arrest during intraoperative cerebral aneurysm rupture. World Neurosurg..

[63] El-Menyar A., Gehani A. (2010). Adenosine-induced tachyarrhythmia and cardiac arrest. Future Cardiol..

[64] Intarakhao P., Thiarawat P., Tewaritrueangsri A., Pojanasupawun S. (2020). Low-dose adenosine-induced transient asystole during intracranial aneurysm surgery. Surg. Neurol. Int..

[65] Fang T.D., Lippmann M., Kakazu C., Donayre C.E., Bui H., Kopchok G.E., White R.A. (2008). High-Dose Adenosine-Induced Asystole Assisting Accurate Deployment of Thoracic Stent Grafts in Conscious Patients. Ann. Vasc. Surg..

[66] Saritas A., Erbas M., Gonen I., Candar M., Ozturk O., Kandis H., Sezen G. (2012). Asystole after the first dose of ceftriaxone. Am. J. Emerg. Med..

[67] Grady E.C., Barron J.T., Wagner R.H. (2011). Development of asystole requiring cardiac resuscitation after the administration of regadenoson in a patient with pulmonary fibrosis receiving n-acetylcysteine. J. Nucl. Cardiol..

[68] Milstein B.B. (1961). Cardiac resuscitation. Br. J. Anaesth..

